# Clinical Prediction of Posttreatment Migraine Recurrence Using Biofeedback Data: A Machine Learning Framework for Enhanced Patient Stratification and Treatment Monitoring

**DOI:** 10.1155/bmri/1282998

**Published:** 2026-03-20

**Authors:** Shibbir Ahmed Arif, Ferdib-Al-Islam, Mehidy Hasan Sium

**Affiliations:** ^1^ School of Computing, Montclair State University, Montclair, New Jersey, USA, montclair.edu; ^2^ Department of Computer Science and Engineering, Northern University of Business and Technology Khulna, Khulna, Bangladesh

**Keywords:** ensemble model, machine learning, outlier detection, permutation feature importance, posttreatment migraine headache, SMOTE

## Abstract

Migraine is a complex neurological disorder with significant implications for individual well‐being and public health. Predicting migraine occurrences after treatment is crucial for evaluating therapeutic efficacy and enabling personalized care, yet remains largely underexplored. This study proposes a robust machine learning framework to predict posttreatment migraine headache occurrences using real‐world headache log data collected from 133 patients undergoing biofeedback therapy. The methodology includes rigorous data preprocessing, outlier removal via the interquartile range (IQR) method, and class imbalance correction through the synthetic minority oversampling technique (SMOTE). A total of 10 classical and a hybrid ensemble machine learning models were developed and optimized through GridSearch with fivefold cross‐validation. Performance was evaluated using different metrics, with the best‐performing hybrid ensemble model achieving an accuracy and F1‐score of 81%, with an area under the receiver operating characteristic curve (AUROC) of 0.87. Additionally, permutation feature importance analysis was employed to enhance model interpretability, identifying medication status, duration of treatment, and patient age as critical predictors. These outcomes validate the prospect of explainable AI‐driven models in forecasting migraine recurrence posttreatment, providing a step forward toward intelligent clinical decision support systems for migraine management.

## 1. Introduction

Migraine headache is a type of neurovascular syndrome that is very frequent and complicated. Until now, it has affected millions of people worldwide and become a significant burden on medical care. According to research, the effect of migraine is enormous and is nearly 10% of the population living in the world, with a greater occurrence specifically among young people and women [1]. According to the Global Classification of Headache Conditions, it is classified as a primary headache disorder that causes mild to severe aching on one side of the head and can be supplemented by various symptoms, including vomiting, nausea, and sensitivity to sound and light [2].

The nature of migraines is complex, involving a combination of environmental, genetic, and physiological factors. Therefore, diagnosis and medication are difficult. Conventional diagnostic strategies are mainly dependent on individual patient reports and diagnostic assessments. In addition, there are wide ranges of migraine symptoms, and so the traditional diagnostic strategy can result in some discrepancy and possible incorrect diagnosis. Hence, there is a strong demand for new methodologies to enrich the precision of migraine diagnosis and forecasting. The need for enhanced precision in migraine diagnosis and forecasting is particularly acute in regions with inadequate medical infrastructure [1].

Despite advancements in machine learning (ML) applications for predicting migraine headaches, several research gaps remain. Existing studies often focus on identifying triggers and predicting migraine onset, but there is limited research on predicting migraine occurrence posttreatment, which is crucial for evaluating treatment efficacy [3]. Many models suffer from class imbalance issues and lack of robustness in handling outliers, leading to a reduced predictive accuracy [4]. Furthermore, most of these studies utilized small and homogeneous datasets, which restrict the generalizability of the discoveries to broader populations [5]. There is also a notable lack of explainability in ML models used for migraine prediction, which poses challenges for clinical integration and patient trust [6]. Addressing these gaps through comprehensive, explainable, and clinically validated ML models could significantly enhance personalized treatment strategies for migraine management.

The key contribution of this paper is a robust ML framework designed to predict posttreatment migraine headache occurrences. This framework addresses the identified limitations by integrating advanced ML techniques with outlier detection and class imbalance handling. The study leverages a real‐world dataset of headache logs from patients undergoing biofeedback treatment, providing insights into the efficacy of predictive models in a clinical setting. Employing SMOTE to address the class imbalance and utilizing the interquartile range (IQR) for outlier detection, the research ensures more accurate and reliable predictions. Additionally, we incorporate the permutation feature importance (PFI) technique to not only enhance model interpretability but also provide clinically actionable insights, thereby facilitating the models′ adoption into clinical practice. The findings emphasize the significance of personalized medicine, highlighting the role of ML in improving healthcare outcomes for migraine patients.

The following parts of the paper are outlined as follows: the “Related Works” illustrates the previous works on migraine prediction using ML. The “Proposed Method” outlines the overall methods applied in this research. The “Result and Discussion” discusses the outcomes of this work, and finally, the “Conclusion” concludes the paper with forthcoming research directions.

## 2. Related Works

In the study, Khan et al. [1] focused on four ML algorithms—support vector machine (SVM), k‐nearest neighbors (KNN), random forest (RF), and decision tree (DT)—along with the deep neural network (DNN) algorithm to classify seven types of migraines. Initially, the models were trained using a publicly available dataset comprising 400 clinical records of patients. Subsequently, the influence of data augmentation was examined, resulting in an increase of patient records to 1447 to evaluate the models′ performance. The findings indicate that the suggested models, utilizing data augmentation, achieve excellent accuracy in diagnosing various types of migraine, with the DNN model attaining a maximum accuracy of 99.66%. The research suggests that these MLs may serve as practical tools for forecasting and diagnosing migraines in patients, particularly in developing nations where medical resources, personnel, and physicians are scarce.

Ullah et al. [7] recommended a hybrid ML model to classify migraine based on the exploration of electroencephalography (EEG) signals. They used a dataset consisting of EEG signals recorded from 21 healthy individuals and 18 migraine patients, obtained under three different stimuli: resting state, visual stimulus, and auditory stimulus. From a total of 144 channels, 14 channels were considered as the data source. They used a total of three cases for this comparative analysis, taking a single‐stimulus approach at a time, a two‐stimulus hybrid approach, and an all–three‐stimuli combined approach. They trained and tested different ML models, where the logistic regression (LR) model with a two‐stimulus hybrid approach performed the best with 99.74% accuracy.

In the study, Orhanbulucu and Latifoğlu [8] developed five different ML models to classify various types of migraine. They utilized a Kaggle public dataset consisting of 400 migraine patient records, where 247 were migraine typical with aura, 80 were migraine without aura, and 73 were other types of migraine. They applied different ML models where the RF algorithm was the most successful model, obtaining accuracy of 95.14%, true positive of 95.10%, false positive of 2.40%, kappa statistics of 92.71%, and mean absolute error of 6.50%.

According to Velgina et al. [9], ML models have been compared to determine the best model with optimal accuracy and precision in predicting the type of migraine based on diverse factors. They trained and tested six ML models on a machine‐generated dataset containing 23 parameters, based on which the algorithm predicts the type of migraine a patient suffers from. The models are finally compared based on 13 evaluation metrics. Among the models, KNN outperformed other models with 85% accuracy and 96% precision.

In the study, Soroor et al. [10] applied five ML models to classify patients with migraines and Tension‐Type Headaches (TTH) based on psychological questionnaires and demographic data completed by 79 patients with migraine headaches and 81 patients with TTH. The Boruta algorithm was applied to select the most relevant features. RF and Naïve Bayes (NB) achieved the highest accuracy, sensitivity, and specificity in distinguishing TTH and migraine.

Anker et al. [11] developed a ML model to predict migraine headaches. Here, 18 migraine patients completed a total of 388 data entries using a headache diary app as well as wearables. In the predictive modeling phase, they included a total of 295 days. They applied a holdout partition of the dataset in which the RF model accomplished the best, with an AUC score of 0.62. Estimating models with high analytical accuracy is likely to be produced by employing a rich dataset of objective biomarkers, self‐reported data, and external data.

In the study, Mitrović et al. [12] applied seven ML models on magnetic resonance imaging (MRI) data to differentiate between healthy persons and those who have suffered from migraine with aura (MwA), as well as between simple and complex forms of MwA. Seventy‐eight individuals took part in this study (32 healthy controls and 46 MwA patients), where cortical features of specific brain regions were found to be essential classification markers. The linear discriminant analysis (LDA) model demonstrated the best performance, achieving 97% accuracy in differentiating MwA patients from healthy controls and 98% accuracy in categorizing between complex and straightforward MwA cases. The conclusions of this study recommend prospective consequences for improving MwA diagnosis and subtype classification, which could affect treatment plans.

Mitrović et al. [13] aim to increase a deeper understanding of the pathophysiology of MwA by investigating the application of ML to structural MRI data for predicting the Migraine Aura Complexity Score (MACS). There were 340 MRI features of 40 MwA patients in the dataset, where each subject contained its average MACS score. They utilized a wrapper feature selection strategy and tested various ML models, including SVM, RBF, and LR. The SVM outclassed the other models, attaining a coefficient of determination of 0.89. The research findings indicate that specific cortical features are linked to MwA complexity, primarily in the temporal and parietal lobes. The proposed method highlights the advantages of ML in predicting MACS scores. It guides the implementation of diagnostic techniques for MwA subtypes and therapies, as well as for future investigations.

In the study [14], Mary et al. developed a sophisticated framework by applying ML models to classify various types of migraines. They optimized feature selection using scatter search and CFS subset assessment to improve classification accuracy. Additionally, they employed the SMOTE to eradicate the concern of class imbalance in the migraine dataset. As a result, the RF performed the best among the ML algorithms, achieving 98.26% accuracy with 400 instances and 24 selected features associated with migraine diagnosis. They further limited the set of features to 18 while still achieving an impressive 98% accuracy. The research demonstrates the significance of feature optimization in improving classification results while also highlighting the efficiency of the RF model in classifying migraine types.

Aung and Srimaharaj [15] presented a computational diagnostic technique that utilizes ML to categorize migraine types based on a patient′s medical history and symptoms. They employed entropy minimization discretization (EMD) in conjunction with a NB classifier to enhance accuracy and effectiveness in migraine diagnosis. The dataset comprises 400 patients′ health records along with several migraine symptoms. The NB model with EMD performed the best, achieving 97.5% accuracy and outperforming the other models they tested.

Seema et al. [16] highlighted the prevalence and acute effects of migraines, emphasizing their global impact. They applied five supervised ML models to classify the types of migraines based on corresponding symptoms. They used a publicly available dataset from Kaggle, which consists of data from 400 patients along with 24 attributes. They selected 16 out of 24 characteristics for the classification task. In results, the NB model achieved the highest mean weighted accuracy (94.2%) among various types of migraines employing the data mining tool, namely Weka. Precisely, NB got 100% accuracy in detecting classes such as typical aura without migraine and migraine without aura. On the contrary, RF performed the worst, specifically for the sporadic hemiplegic migraine class, as the training data were limited.

Fangfang et al. [17] developed a decision support system that applies ML to diagnose primary headaches, differentiating between migraines and TTH. They built the models using SVM, RF, DT, LR, and gradient boosting (GB) on the medical records of 173 patients. The LR model performed the best, achieving 84% accuracy and a 0.90 AUC score.

Zhu and Dave [18] developed a ML model to detect migraines from both biometric and user‐inputted records of individuals. They applied a publicly available dataset including 4580 patients′ records of migraine as well as nonmigraine days. They utilized the SMOTE to mitigate data imbalance and bias. They emphasized employing frequent migraine triggers for predicting incidence, which can be reached from home. The LR model got the best accuracy of 97%, outperforming the RF model. Moreover, they designed a convenient website called MyGraine for evaluating personalized risks.

Despite the promising performance reported in existing migraine prediction studies (in Table 1), several critical limitations remain unresolved. Most prior research has primarily focused on migraine diagnosis, subtype classification, or preonset prediction using highly controlled data sources such as EEG or MRI, rather than forecasting migraine recurrence following therapeutic intervention. Moreover, many studies rely on small or homogeneous datasets, limiting the generalizability of their findings to real‐world clinical settings. Issues related to class imbalance and outlier sensitivity are often insufficiently addressed, which can compromise predictive reliability in longitudinal patient‐reported data. In addition, the majority of existing models function as black boxes, offering limited explainability and thereby restricting clinical trust and adoption. These gaps underscore the need for a robust, explainable ML framework that can reliably predict posttreatment migraine recurrence using real‐world biofeedback data while explicitly addressing data imbalance, outlier robustness, and clinical interpretability—these are the objectives that this study aims to fulfill.

**Table 1:** Review summary.

Reference	Method	Dataset	Findings	Drawbacks
[1]	SVM, KNN, RF, DT, and DNN.	Publicly available dataset from Kaggle containing 400 patients clinical records with seven types of migraines; applied data augmentation that increased the patient records to 1447.	SVM, KNN, DT, and RF with data augmentation achieved 94.60%, 97.10%, 88.20%, and 98.50%, respectively; The DNN model obtained the highest accuracy of 99.66%.	Limited generalizability due to reliance on a small, augmented public dataset; did not explore complex deep learning models or real‐world clinical validation.
[7]	RF, SVM, LR, NB, ANN	EEG signals recorded from 21 healthy and 18 migraine patients obtained in three different stimuli: resting state, visual stimulus, and auditory stimulus; from a total of 144 channels, 14 channels were considered as the data source.	With a two‐stimulus hybrid approach, RF obtained 98.52% accuracy, whereas LR model performed the best with 99.74% accuracy.	Employed a small number of patient records.
[8]	FURIA, KNN, RF, and LibSVM	Publicly available dataset from Kaggle consisting of 400 migraine patient records, where migraine typical with aura were 247, migraine without aura were 80, and other types of migraine were 73.	Utilized SMOTE method to balance the dataset; RF was the best model obtaining accuracy 95.14%, true positive 95.10%, false positive 2.40%, kappa statistics 92.71%, and mean absolute error 6.50%.	Could not compare the results with existing studies as very few studies used same data structures; used a small dataset.
[9]	DT, RF, SVM, NB, KNN, and GB	Publicly available dataset from Kaggle containing 400 rows and 24 columns.	Among the models, KNN outperformed other models with 85% accuracy and 96% precision.	The dataset was small; did not apply deep learning algorithms with real‐world data.
[10]	RF, SVM, LR, NB, and KNN.	Used 160 real‐world patients records, where 79 patients had migraine headache and 81 patients had tension‐type headache (TTH).	Used different questionnaires; applied Boruta algorithm for selecting the most important features; RF, LR, NB, and KNN achieved 98% accuracy and 96% specificity.	Used a small dataset; the generalizability of the models is limited.
[11]	RF, GB, LR, SVM, AB, and XGBoost.	18 migraine patients completed a total of 388 data entries using a headache diary app as well as wearables.	Included 295 days at predictive modeling phase; applied a holdout partition of the dataset in which RF was the top model with an AUC score of 0.62 on the out‐of‐sample test set.	Low predictive performance (*A* *U* *C* = 0.62); small sample size, limiting generalizability.
[12]	LR, LDA, KNN, CART, NB, SVM, and RF.	78 individuals took part in the study, where 32 patients with healthy controls and 46 patients with MwA (22 simple and 24 complex) along with 340 features in total.	The LDA model demonstrated the best performance with 97% accuracy for differentiating MwA patients from healthy controls and 98% accuracy for categorizing in between simple and complex MwA.	Information about the lateralization of MwA attacks is limited; did not use the information of the patients who have only migraines without aura.
[13]	SVM, RBF network, and linear regression	340 MRI features of 40 MwA patients were collected along with the mean MACS score for each patient.	Used a wrapper feature selection method and correlation test for feature selection; SVM outperformed other models with the best 0.89 coefficient of determination.	Used structural MRI data, which might be unable to generalize across different groups and incorporate all related neurological structures; did not explore deep learning architectures; did not employ clinical validation.
[14]	NB, SVM, DT, RF, and MLP.	Data contains 400 instances along with 24 features; seven types of migraine.	Applied SMOTE to deal with the class imbalance issue in the data; the RF model performed the best with the highest 98.26% accuracy for scatter search; further reduced the features to 18 and obtained an impressive 98% accuracy.	Utilized a small dataset; the generalizability of the models is limited.
[15]	NB with EMD, NB without EMD, KNN, J48, DT, RF, and MLP.	Data consists of 400 patients medical records with 23 features; seven migraine types.	Applied tenfold cross validation to verify the models performance; The NB model with EMD performed the best with 97.5% accuracy and 0.98 recall.	The dataset size is small; did not validate in real‐world settings.
[16]	NB, SMO, MLR, J48, DT, and RF.	Used publicly available dataset from Kaggle consisting 400 patients data with 24 features; eight types of migraine.	Selected 16 features; Incorporated Weka tool for implementing ML‐based multiclass classification; The NB model achieved the highest mean weighted accuracy 94.2% using Weka.	Lack of large dataset usages; limited generalizability of the model.
[17]	DT, RF, GB, SVM, and LR.	300 patients with primary headache participated in questionnaires but finally considered 173 patients records with 89 patients having TTH and 84 patients having migraine headaches.	Employed cross‐validation and holdout methods to construct the discriminant models of the primary headache; the logistic regression model achieved the best 84% accuracy and 0.90 AUC score; applied univariate statistical analysis and machine learning for feature selection; performed the chi‐square test to determine 10 most significant features; nausea/vomiting and photophobia/phonophobia could be the two main features to differentiate the two types of disorders.	Small sample size; did not perform real‐time clinical validation.
[18]	LR and RF	Publicly available dataset including 4580 patients records of migraine as well as nonmigraine days.	Utilized SMOTE to reduce the data imbalance and the bias; the Logistic regression model obtained the best accuracy of 97%; emphasized on the number of triggers as the migraines deciding factor; designed a website namely MyGraine for assessing personalized risks.	The model′s generalizability and clinical validation are limited; RF model relies on some specific features for accuracy improvement.

## 3. Proposed Method

### 3.1. System Flowchart

**Figure 1:**
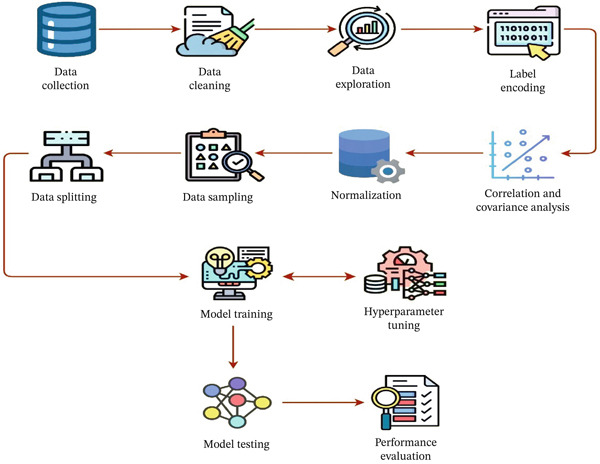
Workflow of the system.

We have visualized the system′s flowchart in Figure 1. The proposed methodology for this study on migraine headache prediction involves a systematic approach starting with dataset preparation, where duplicate values are removed and outliers are identified and dropped using the IQR method. Label encoding is applied to categorical variables, followed by data normalization. To address class imbalance, SMOTE is employed. The dataset has been partitioned into training and testing sets using an 80:20 ratio. Various ML classifiers are applied, with hyperparameter tuning conducted through GridSearch cross validation (fivefold) to optimize model performance. Finally, the models are evaluated with PFI scores computed to interpret the implication of different features in the model′s predictions.

### 3.2. Dataset

We have collected a publicly available dataset from Kaggle [19], which is also available to download via R package [20]. The data are based on headache records completed by 133 patients in a biofeedback‐based treatment program to decrease the intensity and frequency of the attacks of migraine. Over a period of approximately 3 years, patients joined the program at various times. Only 55 patients had data available before the start of treatment, despite being instructed to initiate their records 4 weeks beforehand treatment commencement and to maintain them for 1 month afterwards. The dataset contains 4152 instances and nine features (in Table 2), comprising records from 133 patients.

**Table 2:** Features description.

Feature name	Description
id	Denotes the patients id
Time	Time in days from the start of the treatment, which happens at Time 0.
dos	Time in days from the beginning of the study, since January 1st of the study′s first year of operation.
hatype	Variable that represents the patient′s migraine types, with levels denoting aura, no aura, and mixed.
Age	At the start of treatment, expressed in years.
airq	Measure of the quality of air.
Medication	Variable leveled as none, continuing and reduced. It represents patients who stopped taking the medication, continued their previous dose, or continued at a reduced dose.
Headache	Variable leveled as yes or no.
Sex	The gender of the patients expressed with levels male or female.

### 3.3. Data Cleaning

#### 3.3.1. Null and Duplicate Values

There are no null values in the dataset. However, we have found one duplicate value in the dataset. Therefore, we have dropped it.

### 3.4. Outlier Detection Using IQR

Prior to data normalization and class imbalance handling, outlier detection and removal were performed to improve model robustness and prevent biased learning. The IQR method was employed due to its effectiveness in identifying extreme values in skewed and real‐world clinical datasets.

For each continuous feature, the first quartile (Q1, 25th percentile) and third quartile (Q3, 75th percentile) were computed, and the IQR was defined as the difference between Q3 and Q1. Observations lying outside the lower and upper bounds—calculated as Q1 − 1.5 × IQR and Q3 + 1.5 × IQR, respectively—were considered outliers and removed from the dataset.

In this study, the IQR‐based filtering was applied to the continuous variables “time” and “airq,” as these features exhibited extreme values that could adversely affect model training. After removing outliers from the “time” feature, the dataset was reduced to 3941 samples, and further filtering of the “airq” feature resulted in a final dataset of 3790 samples. This outlier removal step was intentionally performed before normalization and SMOTE oversampling to ensure that synthetic samples were generated from representative data distributions, thereby enhancing the stability and generalizability of the predictive models.

### 3.5. Data Exploration

**Figure 2:**
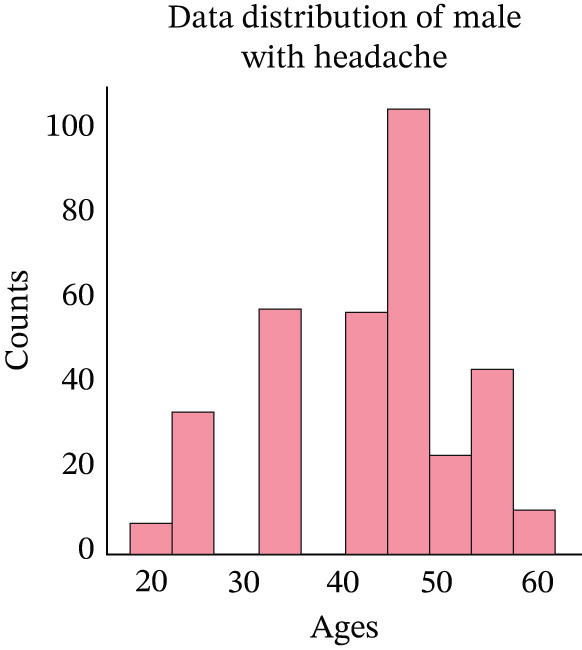
Distribution of headache occurrences among male patients.

**Figure 3:**
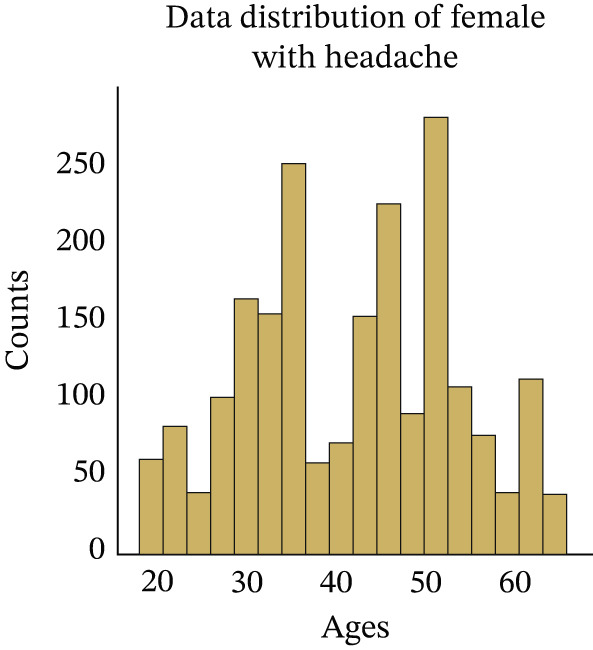
Distribution of headache occurrences among female patients.

**Figure 4:**
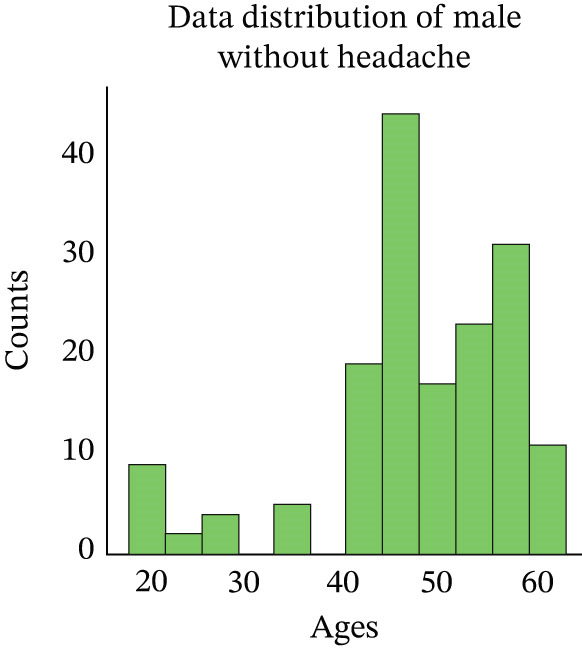
Distribution of male patients without headache.

At this step, we have explored the cleaned data. In Figure 2, we have visualized the data distribution of males with headaches. This figure illustrates the number of males who experience headaches at various age ranges. Then, we have visualized the data distribution of females with headaches in Figure 3. This figure illustrates the number of females who experience headaches at various age ranges. Then, we have visualized the data distribution of males without headaches in Figure 4. This figure indicates the number of males who experience no headaches in different age ranges.

**Figure 5:**
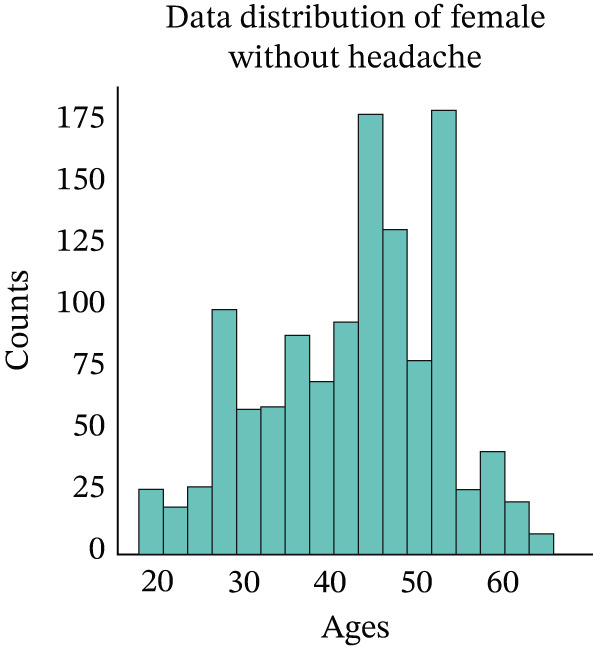
Distribution of female patients without headache.

**Figure 6:**
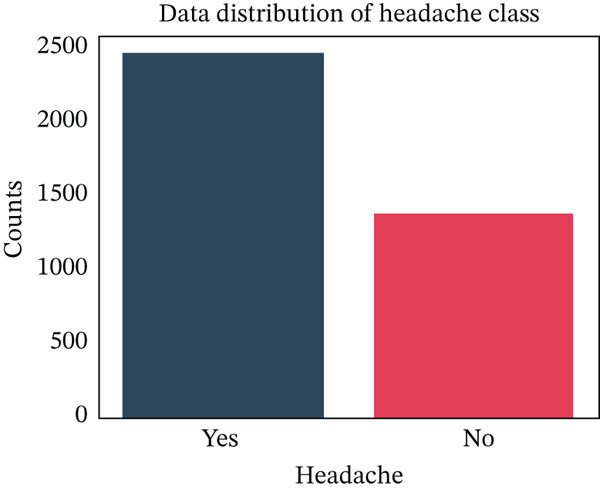
Class‐wise distribution of headache in patients.

Then, we have visualized the data distribution of females without headaches in Figure 5. This figure indicates the number of females who experience no headaches in different age ranges. We then checked the data distribution of headaches, as shown in Figure 6. This figure indicates that approximately 2400 people experience headaches, whereas about 1350 people experience no headaches in the dataset.

### 3.6. Label Encoding

Label encoding was applied to four categorical features in the dataset to convert nonnumeric variables into numerical representations suitable for ML models. These categorical features include: “headache” (binary outcome variable indicating the presence or absence of migraine headache), “sex” (male or female), “hatype” (migraine type categorized as aura, no aura, or mixed), and “medication” (treatment status categorized as none, continuing, or reduced). All remaining variables, including “age,” “time,” “dos” (duration of study in days), and “airq” (air quality measure), were treated as continuous features and therefore were not subjected to label encoding. This clear separation of categorical and continuous variables ensures appropriate preprocessing and prevents unintended distortion of numerical feature distributions.

### 3.7. Data Points Analysis

Here, we have explored the covariance among the variables of the dataset.

**Figure 7:**
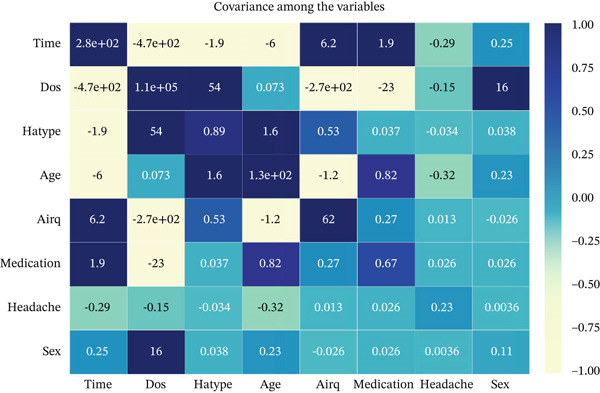
Covariance heat map.

In Figure 7, we can see that the top three pairs of features having high covariance are as follows:

“dos” and “time”: Having a negative covariance of −473.51, these two features show the most significant probability of dropping as one increases.

“airq” and “dos”: There is an opposite relation between these two features which is shown by their negative covariance of −273.38.

“airq” and “time”: These two prefer to rise together as they show a little positive covariance of 6.21. However, they exhibit a weak linear interaction.

After that, we have explored the correlation among the variables.

**Figure 8:**
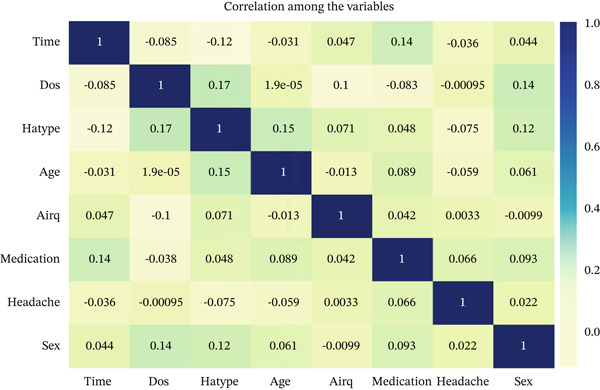
Correlation heat map.

Figure 8 presents the correlation heat map illustrating pairwise linear associations among the dataset variables. Overall, the observed correlation coefficients are weak‐to‐moderate in magnitude, which is typical for complex biological and clinical data where outcomes are influenced by multiple interacting factors rather than strong linear dependencies. Among the features, “dos” and “hatype” exhibit the highest positive correlation (*r* = 0.172), indicating a weak but statistically meaningful association, where an increase in the duration of study is weakly correlated with changes in migraine type classification. Similarly, “age” and “hatype” demonstrate a weak positive correlation (*r* = 0.152), suggesting a modest relationship between patient age and migraine subtype. The features “sex” and “dos” also show a weak positive correlation (*r* = 0.144), implying limited linear association. Although these correlations are not strong in absolute terms, their presence supports the multifactorial nature of migraine recurrence and justifies the use of nonlinear and ensemble of ML models capable of capturing complex feature interactions beyond simple linear relationships.

### 3.8. Normalization

We transformed the data points to a standard range using normalization. It involves scaling the features to a specified range, usually between 0 and 1. The following formula is applied to complete this process:

Xnormalized=X−XminXmax−Xmin

Here, *X*_normalized_ is the normalized value, *X* is the original value, *X*_max_ is the maximum value, and *X*_min_ is the minimum value.

### 3.9. Data Sampling

**Figure 9:**
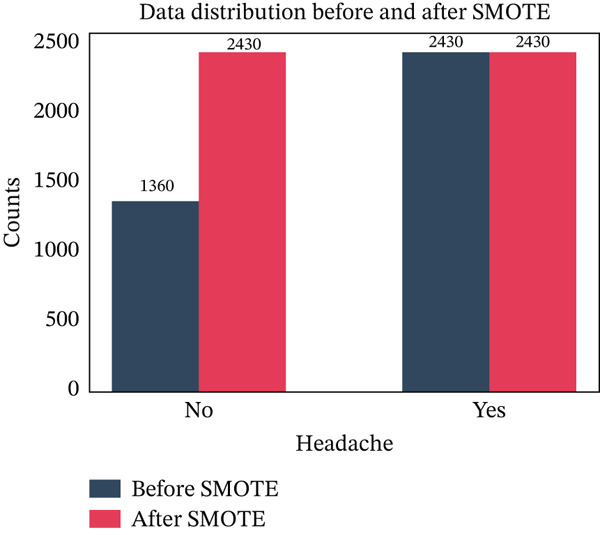
Comparison of class‐wise data distribution before and after applying SMOTE.

As we can see in Figure 9, the target variable “headache” has two classes, named “yes” and “no”, where both have different numbers of data points. Therefore, the classes are imbalanced. At this point, we have applied the SMOTE oversampling technique to remove the class imbalance.

It identifies and targets the minority class named “no”, which has 1360 samples, in comparison with the majority class “yes”, which has 2430 samples. SMOTE determines the KNN of every sample in the minority class. Interpolating between the specific sample and its neighbors, SMOTE generates synthetic samples. To execute the interpolation, a neighbor is selected at random, and an entirely new sample is generated along the segment of the line between the instance and the neighbor. The formula applied to complete this process is as follows:

Synthetic sample=Original sample+λ×Neighbor−Original Sample

Here, *λ* is an arbitrary number that ranges from 0 to 1. By this process, SMOTE generates a sufficient number of synthetic samples in the minority class to make the classes equal.

**Figure 10:**
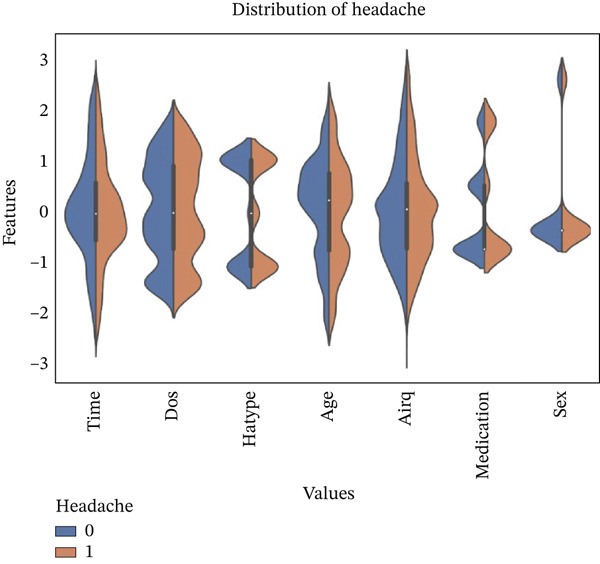
Violin plot depicting the data distribution of headache with associated features.

Figure 10 provides a comprehensive visualization of how the target variable “headache” is distributed in relation to other features in the dataset. The chart reveals that patients reporting headaches (“yes” class) are more frequent than those without headaches (“no” class), which initially led to class imbalance in the dataset. Notably, there appears to be a higher concentration of headache occurrences among individuals who continued their medication regimen and within specific age groups, particularly among younger adults. Additionally, environmental factors such as lower air quality (airq) seem to correspond with increased reports of headaches, suggesting potential external triggers. This distribution highlights important associations that are later confirmed through PFI analysis, where medication status, age, and air quality emerged as significant predictors. The figure effectively demonstrates the complexity and variability in headache patterns, emphasizing the need for robust predictive modeling using advanced ML techniques.

### 3.10. Data Splitting

We have split the data into an 80:20 ratio by applying the *train_test_split*() method available in the Scikit‐learn library to obtain train and test data subsets.

### 3.11. ML Models

We utilized 10 well‐established ML classifiers to predict migraine occurrences after treatment. These models were selected based on their demonstrated performance in medical data analysis and were evaluated using a unified pipeline involving SMOTE, hyperparameter tuning, and fivefold cross‐validation.

#### 3.11.1. SVM

SVM builds a hyperplane that exploits the margin between classes in a high‐dimensional space. It is effective for both linear and nonlinear classification, especially in smaller datasets [21].

#### 3.11.2. NB

NB is a probabilistic model that applies Bayes′ theorem with strong (naïve) independence expectations among the features. It is computationally fast and useful for benchmarking [22].

#### 3.11.3. RF

RF is an ensemble of DTs trained on bootstrapped datasets with random feature selection. It provides robustness to overfitting and handles high‐dimensional data effectively [23].

#### 3.11.4. LR

LR is a linear model that assesses the probability of a binary outcome using the logistic function. It served as a baseline due to its interpretability and efficiency [24].

#### 3.11.5. KNN

KNN is a nonparametric model that classifies samples based on the majority vote of their KNN. It is sensitive to feature scaling and works best in low‐dimensional settings [25].

#### 3.11.6. DT

**Figure 11:**
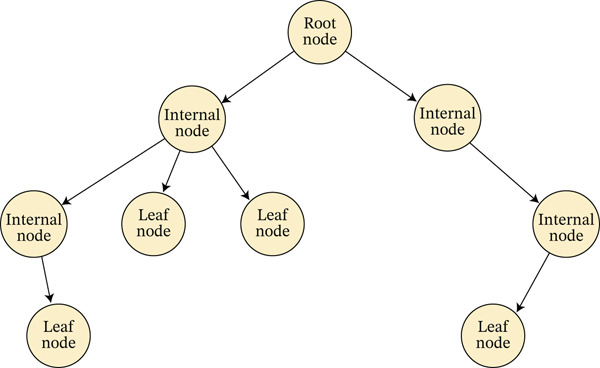
Decision tree.

DT uses a hierarchical structure of feature‐based splits to make decisions. It is interpretable and easy to visualize but prone to overfitting on noisy data [17]. Figure 11 illustrates a simple DT.

#### 3.11.7. GB

GB builds models sequentially by optimizing a loss function through gradient descent. It tends to achieve high accuracy but entails careful hyperparameter tuning to avoid overfitting [26].

#### 3.11.8. LightGBM

LightGBM is a fast, scalable GB framework that uses histogram‐based methods and leaf‐wise tree growth. It is well‐suited for large‐scale and high‐dimensional datasets [27].

#### 3.11.9. XGBoost

XGBoost is a regularized, efficient GB model that improves performance through advanced features like parallel processing and sparsity‐aware learning. It is widely used in ML research and structured data tasks [28].

#### 3.11.10. MLP

**Figure 12:**
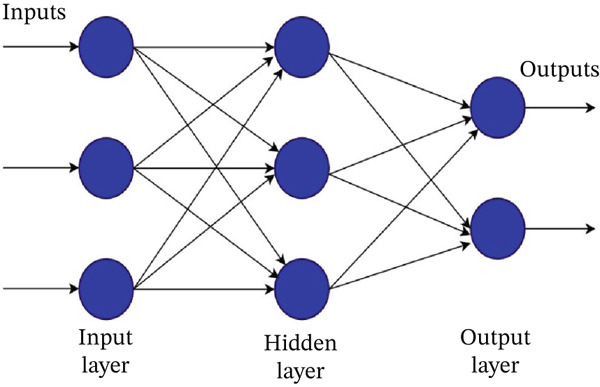
Multilayer perceptron.

MLP is a feedforward ANN, having input, hidden, and output layers. It captures complex nonlinear relationships but requires more computational resources and parameter tuning than traditional ML models [29]. Figure 12 illustrates three inputs, three input nodes, three hidden layers consisting of three nodes, and two output layers having two output nodes.

#### 3.11.11. Hyperparameter Tuning

Hyperparameter tuning was conducted using the GridSearchCV method with a fixed fivefold cross‐validation (*C* *V* = 5) strategy applied uniformly across all ML models. This consistent cross‐validation scheme ensured fair model comparison and reduced variability in performance estimation. Following optimization, each model was retrained using the best‐performing parameter configuration identified through the GridSearch process. Table 3 presents the tuned hyperparameters with their conforming values.

**Table 3:** List of hyperparameter search ranges and optimized values.

**Model**	**Hyperparameter**	**Search Range**	**Optimized Value**	**CV Fold**
Logistic Regression (LR)	C	{0.01, 0.1, 1, 10, 100}	0.1	5
	max_iter	{100, 300, 500}	100	
	penalty	{l1, l2}	l1	
	solver	{saga, liblinear}	saga	

Support Vector Machine (SVM)	C	{1, 10, 100, 1000}	1000	5
	gamma	{0.01, 0.1, 0.5, 0.9, scale}	0.9	
	kernel	{linear, rbf}	rbf	
	probability	{True}	True	

Decision Tree (DT)	max_leaf_nodes	{20, 50, 80, 98}	98	5
	min_samples_split	{2, 4, 6, 10}	4	

K‐Nearest Neighbor (KNN)	n_neighbors	{5, 7, 9, 10, 11}	10	5
	weights	{uniform, distance}	distance	
	metric	{euclidean, minkowski}	minkowski	
	p	{1, 2}	1	
	leaf_size	{10, 20, 30}	20	

Naïve Bayes (NB)	var_smoothing	{1e‐9, 1e‐4, 1e‐2, 1e‐1}	0.001	5

Multi‐Layer Perceptron (MLP)	hidden_layer_sizes	{(50,), (100,), (50,100,50)}	(50,100,50)	5
	activation	{relu, tanh}	relu	
	alpha	{0.0001, 0.001, 0.01}	0.0001	
	learning_rate	{constant, adaptive}	adaptive	
	solver	{adam}	adam	

Random Forest (RF)	n_estimators	{100, 200, 500}	100	5
	max_depth	{20, 50, 80}	80	
	max_features	{sqrt, log2}	log2	
	min_samples_leaf	{1, 2, 3}	3	
	min_samples_split	{2, 5, 8}	8	
	bootstrap	{True}	True	

Gradient Boosting (GB)	learning_rate	{0.01, 0.05, 0.1}	0.1	5
	max_depth	{3, 5, 8}	8	
	min_samples_leaf	{5, 10}	10	
	min_samples_split	{5, 10}	10	
	max_features	{sqrt, log2}	sqrt	
	subsample	{0.8, 1.0}	1.0	

Light Gradient Boosting Machine (LightGBM)	scale_pos_weight	{1, 2, 5}	1	5

Extreme Gradient Boosting (XGBoost)	max_depth	{3, 5, 7}	5	5
	eta	{0.01, 0.05, 0.1}	0.01	
	gamma	{0, 0.05, 0.1, 0.11}	0.11	
	lambda	{0.01, 0.1, 1}	0.01	
	alpha	{0, 0.1}	0	
	min_child_weight	{1, 3}	1	
	subsample	{0.8, 1.0}	1	

Hyperparameter tuning was conducted on the training dataset only, following data preprocessing, outlier removal, normalization, and SMOTE‐based class balancing. The computational cost of the GridSearch procedure was moderate, with total execution time ranging from several minutes for simpler models (e.g., LR and NB) to under 1 h for more complex ensemble and neural network models, depending on the size of the parameter grid. All experiments were executed on a Google Colab environment.

#### 3.11.12. Ensemble of Models

We have utilized the voting classifier with soft voting to combine the best‐trained models and constructed a hybrid ensemble model, thereby achieving better prediction results. In this step, we used the trained models of RF, KNN, GB, and LightGBM, respectively, with fivefold cross‐validation.

## 4. Results and Discussion

In this part, we have visualized the confusion matrices for all the models and then, we have utilized several performance metrics to evaluate the performance of the models.

### 4.1. Confusion Matrix (CM)

Table 4 demonstrates a CM in which “yes” is referred to as “migraine headache exists,” and “no” is referred to as “migraine headache does not exist” [30].

**Table 4:** Confusion matrix.

	Predicted value	
Actual value	Yes	No
Yes	TP	FN
No	FP	TN

The CM comprises four main properties (numbers) that form the evaluating metrics of the model. The four properties are as follows:

1. **TP:** TP stands for true positive. Indicates the total number of patients who have been correctly categorized as migraine headache.
2. **TN:** TN stands for true negative. Indicates the total number of patients who have been correctly categorized as having no migraine headache
3. **FP:** FP stands for false positive. Indicates the total number of patients who have been incorrectly categorized as migraine headache but do not actually have migraine headache.
4. **FN:** FN stands for false negative. It indicates the total number of patients who have been incorrectly categorized as having no migraine headache but actually they are struggling with migraine headaches.

**Figure 13:**
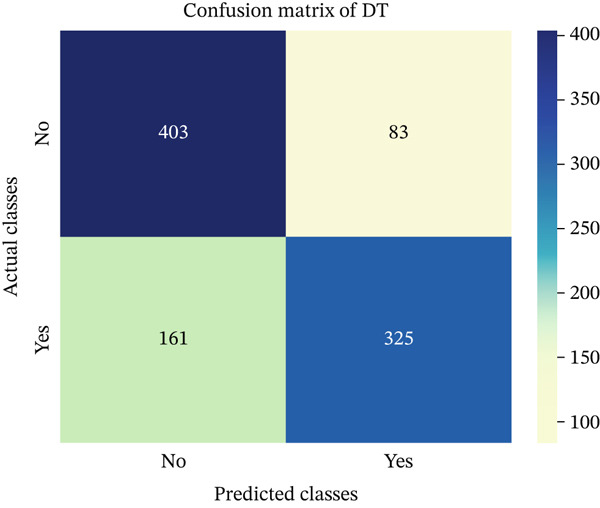
CM of DT model.

**Figure 14:**
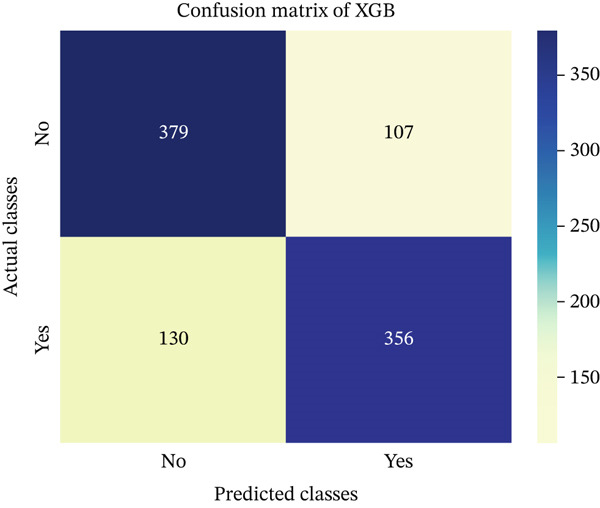
CM of XGBoost model.

**Figure 15:**
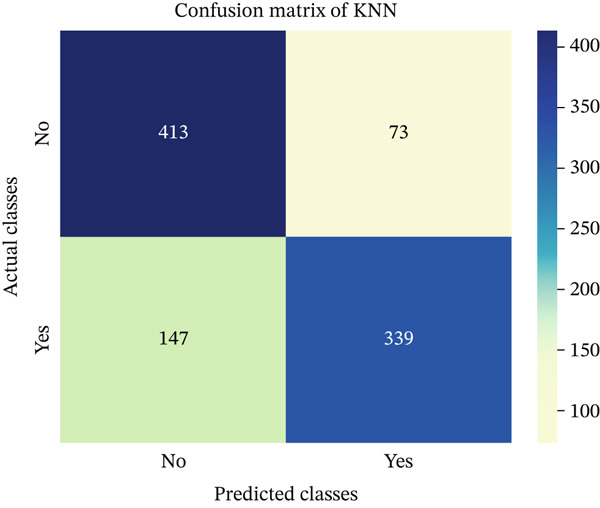
CM of KNN model.

**Figure 16:**
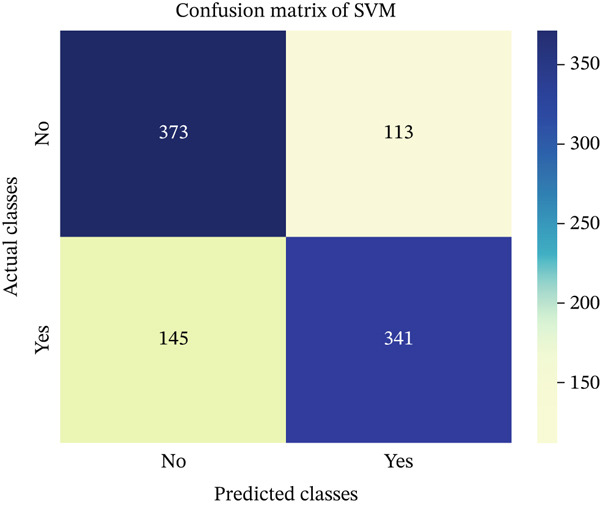
CM of SVM model.

**Figure 17:**
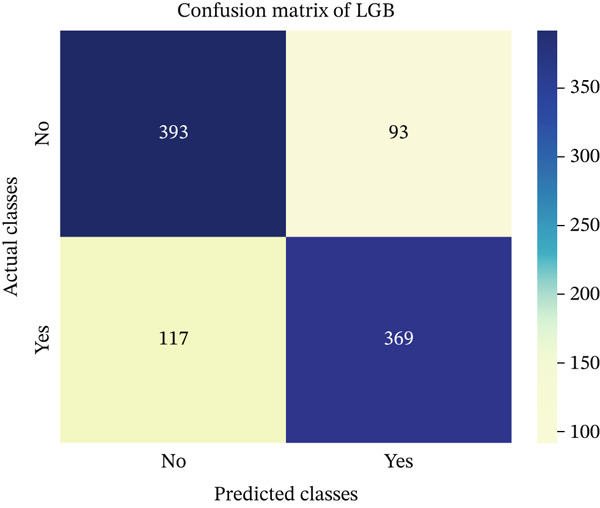
CM of LightGBM model.

**Figure 18:**
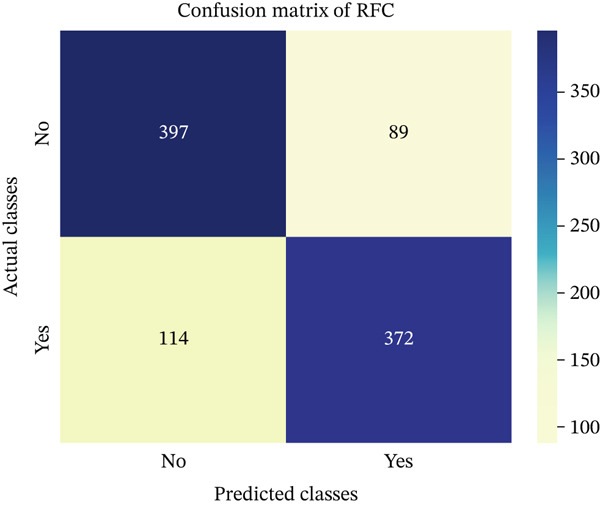
CM of RF model.

**Figure 19:**
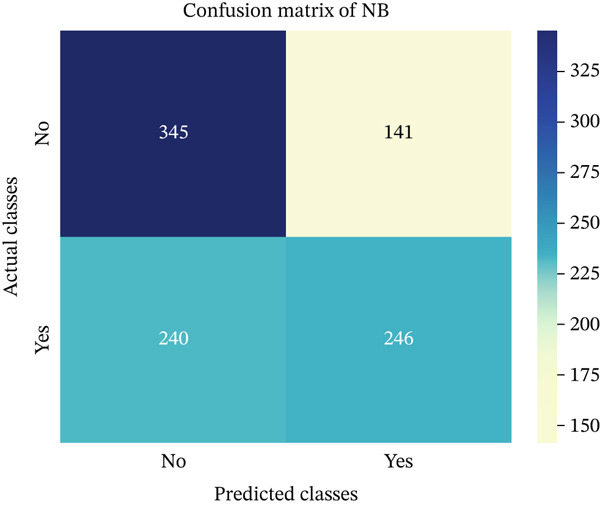
CM of NB model.

**Figure 20:**
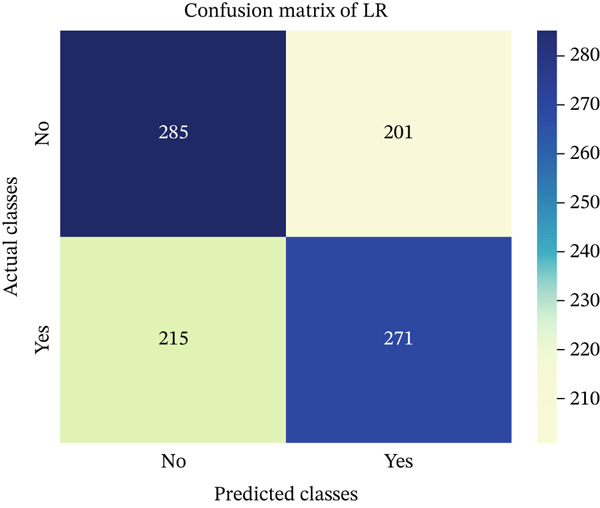
CM of LR model.

**Figure 21:**
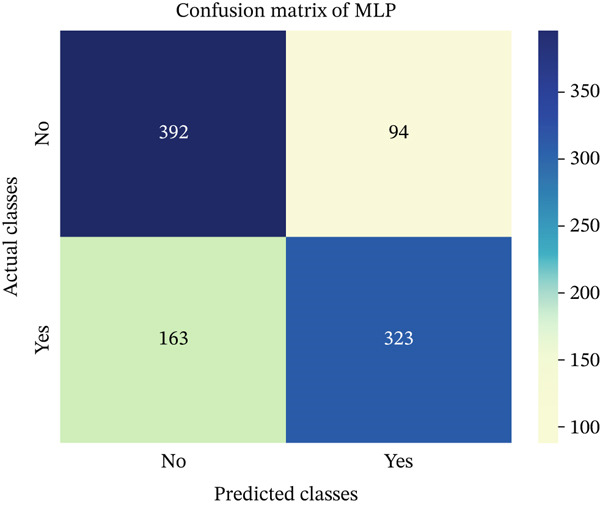
CM of MLP model.

**Figure 22:**
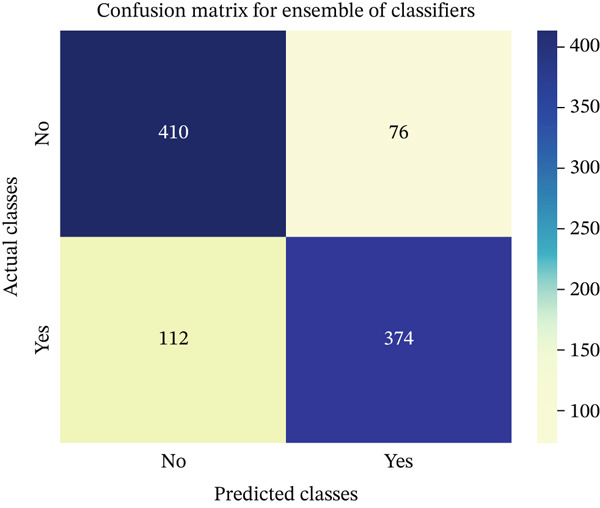
CM of ensemble of models.

Figures 13, 14, 15, 16, 17, 18, 19, 20, 21, and 22 illustrate the CM for each of the ML models applied in this study, providing a detailed view of their classification performance on the test dataset. The diagonal dominance observed in most matrices indicates that the majority of samples were correctly classified into their respective categories—either “yes” (migraine headache) or “no” (no migraine headache). Among the models, the hybrid ensemble classifier (Figure 22) demonstrates the most balanced and accurate classification, with high true positives and true negatives and relatively fewer misclassifications (FP and FN). In contrast, models like LR (Figure 20) and NB (Figure 19) exhibit higher misclassification rates, particularly in failing to detect true negatives, suggesting weaker generalization. The confusion matrices also help identify the cost of false predictions—for instance, a false negative (classifying a migraine patient as headache‐free) is clinically more concerning and observed more frequently in less robust models. These visual diagnostics reinforce the superiority of the ensemble approach while highlighting performance disparities across classifiers, underscoring the importance of model selection in health prediction tasks.

### 4.2. Performance Metrics

The performance metrics that we applied to complete our analysis are accuracy, precision, recall, and F1‐score.

#### 4.2.1. Accuracy

The accuracy of a model is calculated as the proportion of properly identified patients to the total number of patients.

Accuracy=TP+TNTP+FP+TN+FN

#### 4.2.2. Precision

The precision of a model is measured as the proportion of properly identified patients with the migraine headache to the total number of patients expected to have migraine headaches.

Precision=TPTP+FP

#### 4.2.3. Recall

The recall of a model is measured as the proportion of properly identified migraine patients to the total number of patients who actually suffer from migraine.

Recall=TPTP+FN

#### 4.2.4. F1‐Score

The F1‐score determines the point of balance between precision and recall.

F1−score=2∗Precision∗RecallPrecision+Recall

Table 5 presents the classification performance of all applied ML models using their default hyperparameters, providing a baseline for comparison. Among the models evaluated, the RF and LightGBM achieved the highest accuracy of 78%, closely tailed by KNN scoring 76%. However, several models, such as NB, LR, and DT, demonstrated comparatively lower performance, with accuracy ranging between 60% and 72% and lower F1‐scores, reflecting suboptimal generalization and limited predictive power. The area under the receiver operating characteristic curve (AUROC) also varied significantly across models, indicating discrepancies in sensitivity and specificity trade‐offs. These results underscore the importance of hyperparameter optimization, as default settings may not capture the complex feature interactions and distributional nuances present in medical datasets, especially those with class imbalance and small sample sizes. Overall, Table 5 establishes a crucial benchmark to measure the impact of tuning strategies and ensemble modeling in subsequent experiments.

**Table 5:** Performance with default parameters.

Model	Accuracy	Precision	Recall	F1‐score
LR	0.55	0.55	0.55	0.55
DT	0.72	0.72	0.72	0.72
SVM	0.66	0.67	0.66	0.66
NB	0.60	0.61	0.60	0.60
KNN	0.76	0.76	0.76	0.76
GB	0.73	0.73	0.73	0.73
RF	0.78	0.78	0.78	0.78
LightGBM	0.78	0.78	0.78	0.78
XGBoost	0.73	0.73	0.73	0.73
MLP	0.70	0.70	0.70	0.70

Table 6 summarizes the improved classification performance of each model following hyperparameter optimization using GridSearch with fivefold cross‐validation. Notably, nearly all models experienced measurable performance gains in all metrics. The most significant improvements were observed in GB and the hybrid ensemble model (using RF, LightGBM, GB, and KNN), while the hybrid ensemble model achieved the best accuracy of 81% and an AUROC of 0.87, indicating excellent discriminative ability and well‐balanced sensitivity and specificity. In contrast to the baseline results in Table 5, even previously underperforming models such as LR and NB showed moderate gains, though still trailing behind the top‐performing classifiers. These results confirm that hyperparameter tuning substantially enhances model robustness and generalizability, especially in small, imbalanced medical datasets. The improvements also highlight the critical role of model selection and optimization in achieving clinically relevant prediction accuracy, reinforcing the suitability of ensemble learning approaches for migraine outcome forecasting.

**Table 6:** Performance after hyperparameters′ tuning.

Model	Accuracy	Precision	Recall	F1‐score
SVM	0.73	0.74	0.73	0.73
NB	0.61	0.61	0.61	0.60
LR	0.56	0.56	0.56	0.56
KNN	0.77	0.78	0.77	0.77
DT	0.75	0.76	0.75	0.75
RF	0.78	0.78	0.78	0.78
GB	0.78	0.78	0.78	0.78
LightGBM	0.78	0.78	0.78	0.78
XGBoost	0.76	0.76	0.76	0.76
MLP	0.74	0.74	0.74	0.73
Hybrid ensemble model (RF + KNN + GB + LGBM)	0.81	0.81	0.81	0.81

### 4.3. Receiver Operating Characteristic (ROC) Curve Analysis

**Figure 23:**
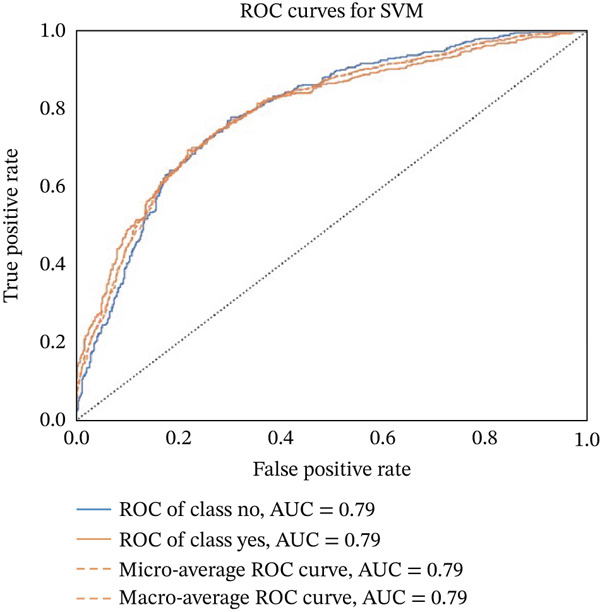
ROC curve for evaluating the SVM model′s classification performance.

**Figure 24:**
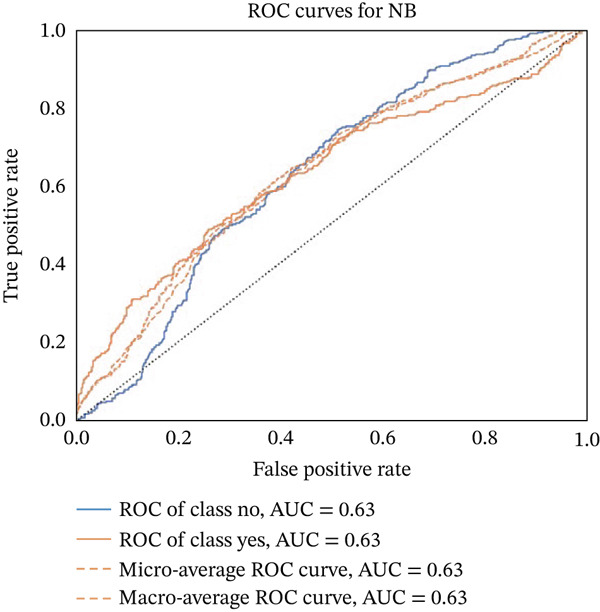
ROC curve for evaluating the NB model′s classification performance.

**Figure 25:**
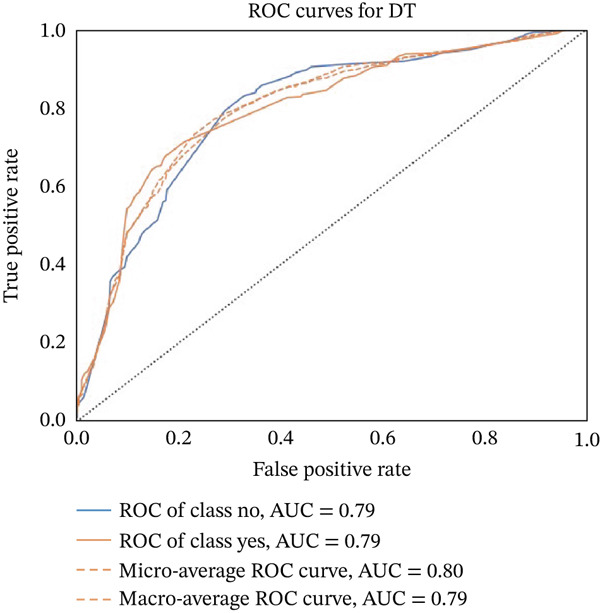
ROC curve for evaluating the DT model′s classification performance.

**Figure 26:**
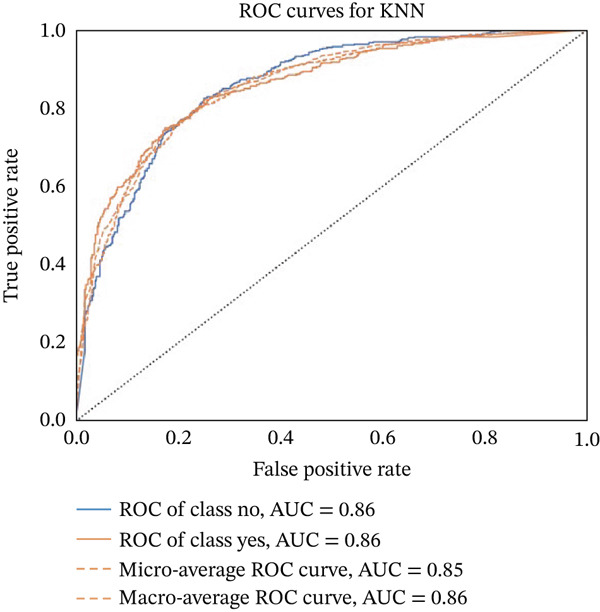
ROC curve for evaluating the KNN model′s classification performance.

**Figure 27:**
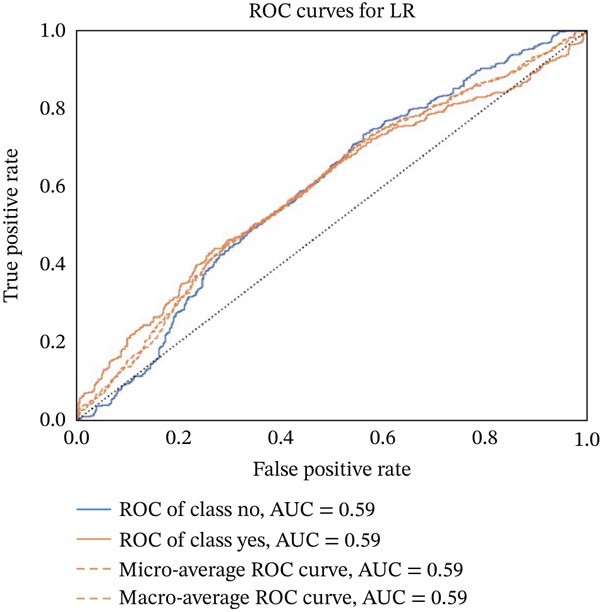
ROC curve for evaluating the LR model′s classification performance.

**Figure 28:**
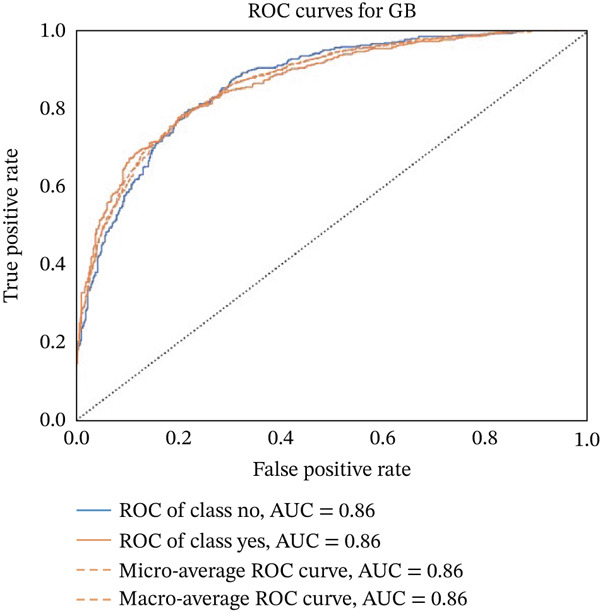
ROC curve for evaluating the GB model′s classification performance.

**Figure 29:**
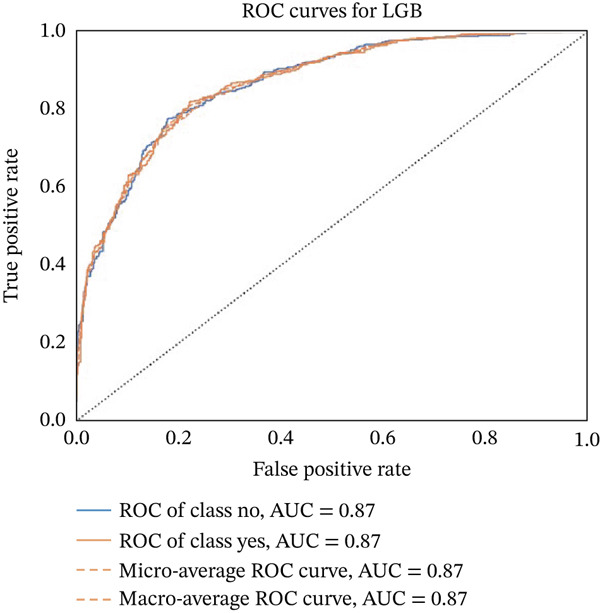
ROC curve for evaluating the LightGBM model′s classification performance.

**Figure 30:**
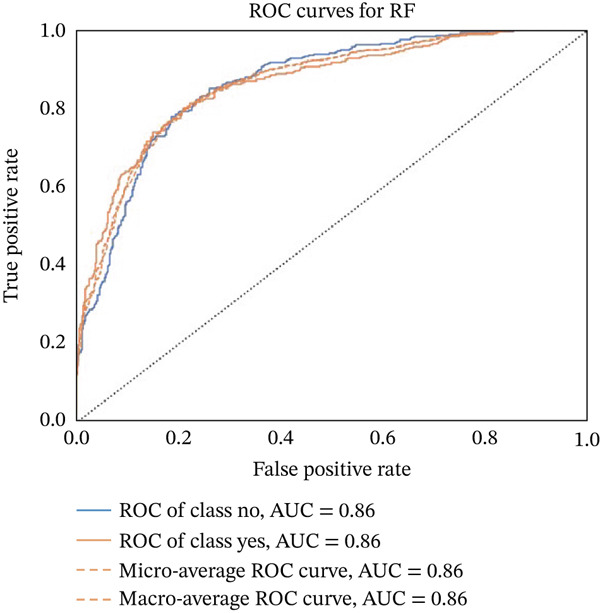
ROC curve for evaluating the RF model′s classification performance.

**Figure 31:**
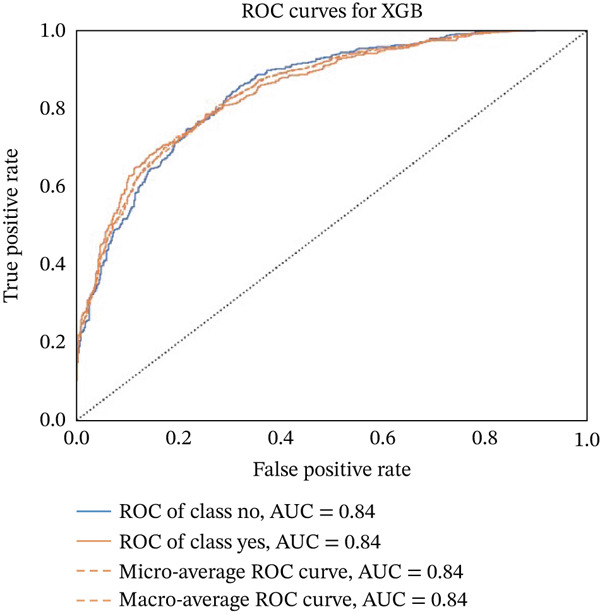
ROC curve for evaluating the XGBoost model′s classification performance.

**Figure 32:**
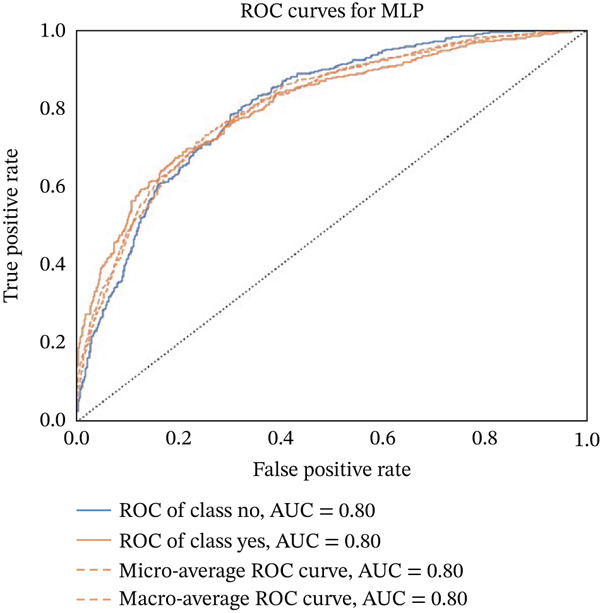
ROC curve for evaluating the MLP model′s classification performance.

**Figure 33:**
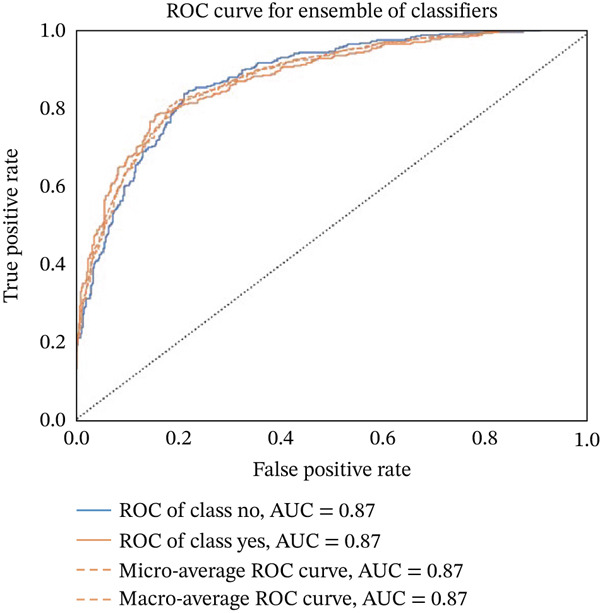
ROC curve for evaluating the ensemble of models classification performance.

**Figure 34:**
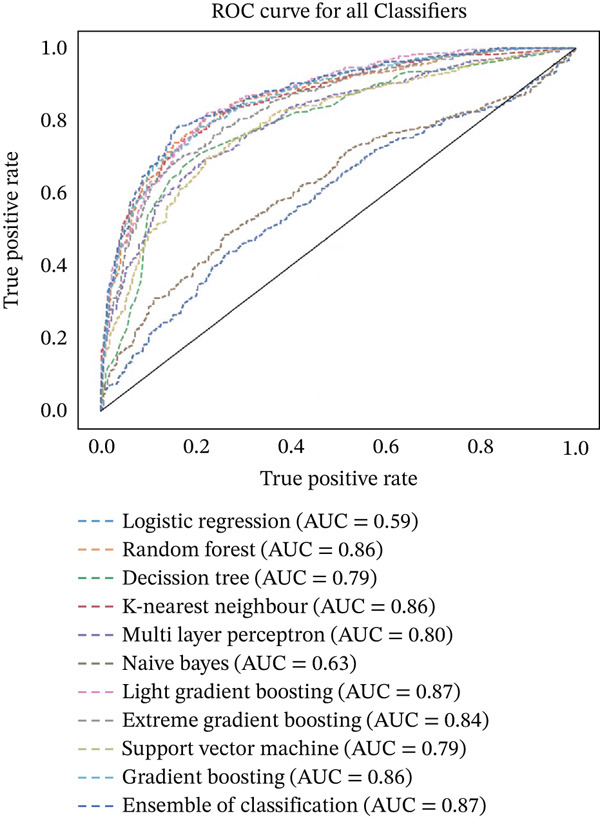
ROC curve illustrating the classification performance across all models.

Figures 23, 24, 25, 26, 27, 28, 29, 30, 31, 32, 33, and 34 depict the ROC curves for each ML model, illustrating their ability to discriminate between migraine and nonmigraine cases. The ROC curve evaluates a model′s performance by plotting the TPR (sensitivity) against the FPR (1—specificity). A curve that bows toward the top‐left corner indicates strong performance. Among all the models, the hybrid ensemble model (Figure 33) and LightGBM (Figure 29) exhibit the best separation between classes, achieving the highest AUROC of 0.87, suggesting excellent classification capability. Other models such as RF, GB, and KNN also demonstrate competitive AUROC values ranging from 0.79 to 0.86, indicating reliable predictive power. In contrast, LR (Figure 27) yields a relatively flatter curve with an AUROC of 0.59, reflecting poor discrimination between classes. Overall, the ROC curves confirm the advantage of ensemble learning in this context and validate the effectiveness of model tuning and feature engineering steps adopted in the proposed framework.

### 4.4. PFI Scores Analysis

**Figure 35:**
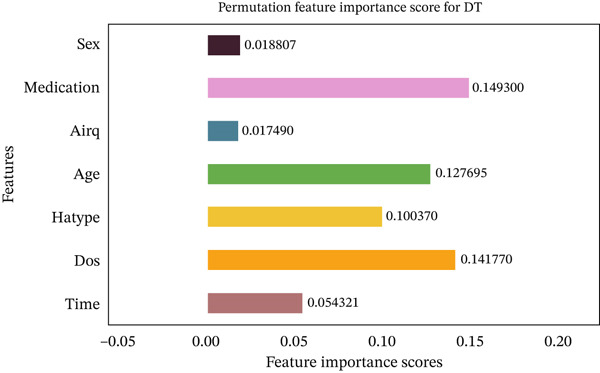
Feature importance scores calculated using permutation for the DT model.

**Figure 36:**
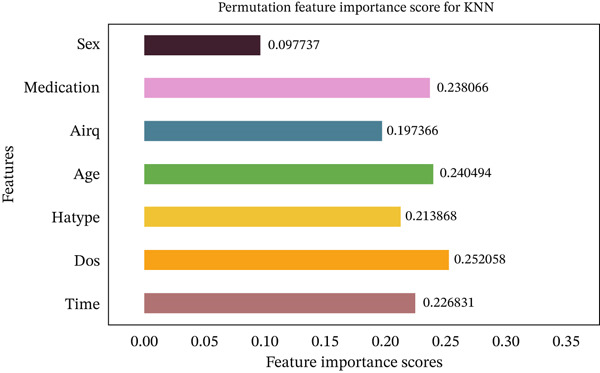
Feature importance scores calculated using permutation for the KNN model.

**Figure 37:**
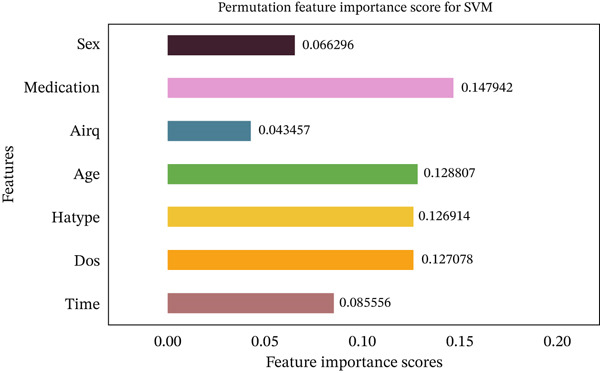
Feature importance scores calculated using permutation for the SVM model.

**Figure 38:**
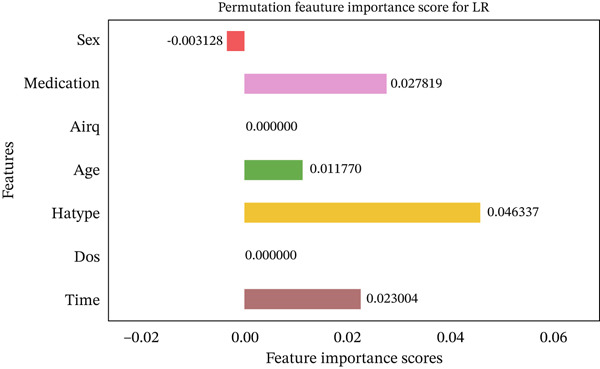
Feature importance scores calculated using permutation for the LR model.

**Figure 39:**
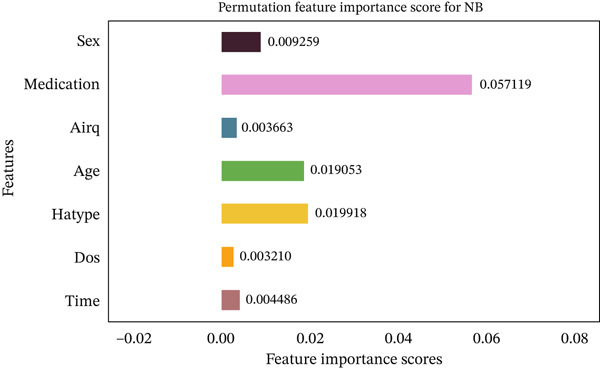
Feature importance scores calculated using permutation for the NB model.

**Figure 40:**
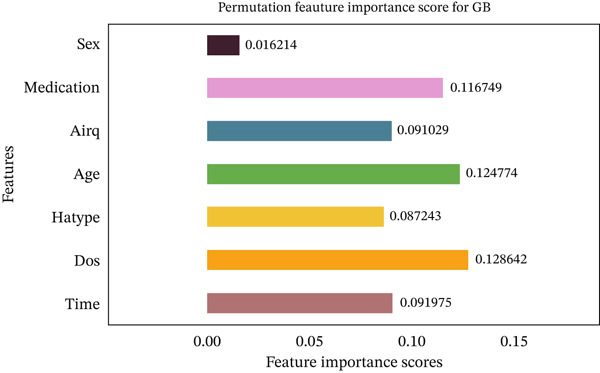
Feature importance scores calculated using permutation for the GB model.

**Figure 41:**
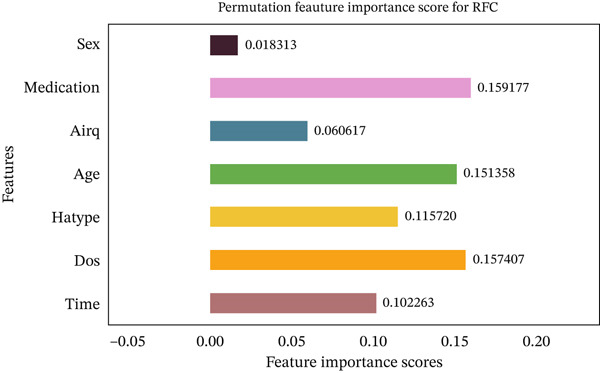
Feature importance scores calculated using permutation for the RF model.

**Figure 42:**
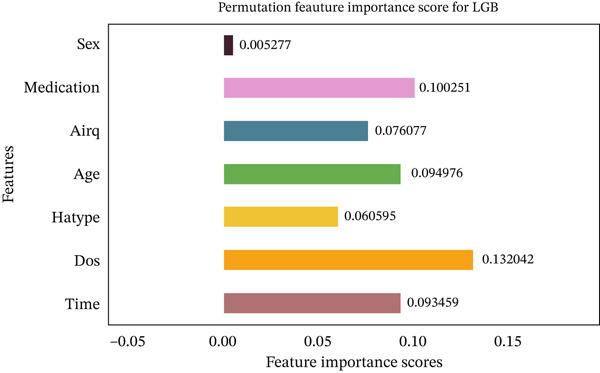
Feature importance scores calculated using permutation for the LightGBM model.

**Figure 43:**
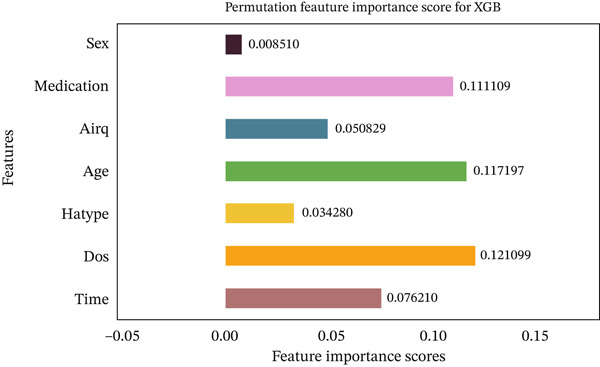
Feature importance scores calculated using permutation for the XGBoost model.

**Figure 44:**
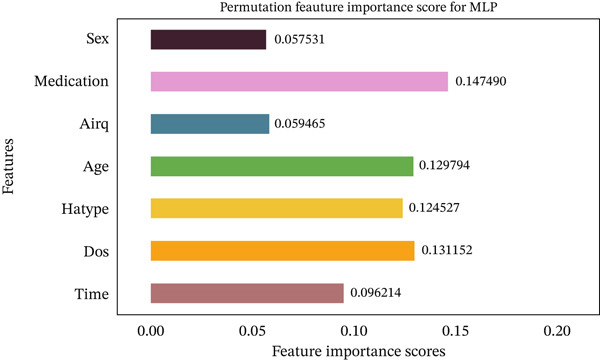
Feature importance scores calculated using permutation for the MLP model.

**Figure 45:**
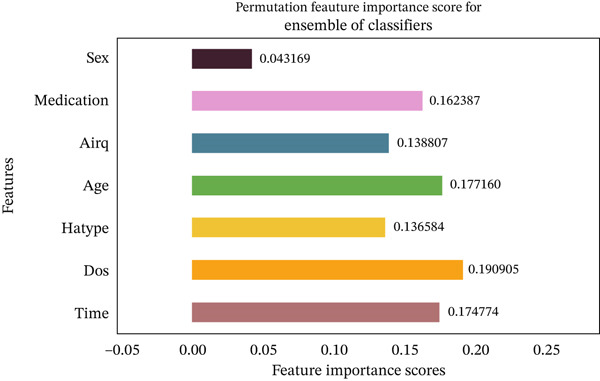
Feature importance scores calculated using permutation for the ensemble of models.

Figures 35, 36, 37, 38, 39, 40, 41, 42, 43, 44, and 45 present the PFI scores for all applied ML models, highlighting the relative contribution of each input feature to the predictive performance. Across nearly all models, certain features consistently emerged as the most influential—specifically, medication status, duration of study (dos), patient age, and time from treatment start. For example, in models such as DT (Figure 35), RF (Figure 41), and the hybrid ensemble model (Figure 45), these four features displayed the highest importance scores, indicating a strong association with the likelihood of posttreatment migraine recurrence. This consistency demonstrates the robustness of these predictors across diverse algorithmic architectures. The inclusion of domain‐relevant features such as medication status (categorized as continuing, reduced, or none) enhances the clinical interpretability of the model′s outputs, which is critical for real‐world deployment. On the other hand, features like “sex” and “air quality” showed comparatively lower importance across most models, suggesting limited influence in this dataset. These insights not only reinforce the validity of the selected predictors but also support the model′s explainability—making it more trustworthy and actionable in a healthcare context.

### 4.5. Discussion

Predicting migraine recurrence posttreatment is crucial for evaluating therapeutic efficacy, yet remains an area with minimal dedicated research [3, 4]. Additionally, class imbalance and a lack of robustness in handling outliers are two significant issues that reduce the prediction accuracy of models in various studies [31]. Furthermore, the generalizability of the findings from most of these studies is limited to broader populations due to the use of small and homogeneous datasets [32]. In our research, we have provided insights into the efficiency of the predictive models in a clinical setting by leveraging a real‐world dataset of headache logs from patients undergoing biofeedback treatment. Moreover, we have integrated advanced ML techniques to predict migraine headache occurrences posttreatment by detecting outliers and addressing class imbalance issues. To ensure more reliable and accurate predictions, we have utilized the IQR method to detect outliers and employed SMOTE to address class imbalance effectively. Additionally, we have provided an extensive analysis to enhance the impact of the features on ML models by incorporating the PFI technique, which can facilitate their potential adoption in clinical practice.

Compared with existing literature, our optimized Hybrid Ensemble Model demonstrates competitive performance, achieving an F1‐score of 81% with an AUROC of 0.87, indicating strong discriminative ability and balanced precision–recall performance. For example, Ullah et al. [7] utilized EEG signals with LR to classify migraine cases, reporting an accuracy of 99.74%. However, their work relied on a highly controlled, stimulus‐based dataset that may not generalize to broader clinical environments. Similarly, studies using MRI‐based features, such as Mitrović et al. [12], have shown high accuracy (up to 97%), yet such imaging modalities are costly and less accessible in low‐resource settings. In contrast, our approach leverages routinely collected biofeedback logs, offering a more practical and scalable alternative for real‐world deployment. Despite using a modest dataset, the combination of careful preprocessing, class balancing, and model optimization yielded reliable and interpretable predictions. Unlike many prior works, we also incorporate explainability (via PFI), which increases model interpretability—an essential criterion for clinical acceptance. Therefore, our findings not only reinforce the utility of classical and ensemble ML techniques in migraine prediction but also showcase their applicability in real‐world, posttreatment monitoring scenarios.

The proposed ML framework can serve as a valuable clinical decision‐support tool by helping healthcare providers predict the likelihood of migraine recurrence after treatment. Such predictive insights can guide clinicians in tailoring individualized follow‐up strategies, medication adjustments, and behavioral interventions for patients with migraines. Furthermore, the model′s explainability through the PFI ensures transparency, which is essential for integration into clinical workflows and gaining trust from medical professionals.

## 5. Conclusion

This study addressed the critical gap in posttreatment migraine recurrence prediction by developing a robust and explainable ML framework using real‐world biofeedback data. We demonstrated that integrating specialized data preprocessing techniques, particularly IQR‐based outlier detection and SMOTE‐based class imbalance handling, is essential for constructing reliable and generalizable predictive models in this complex clinical domain. By focusing on posttreatment outcomes rather than diagnosis or preonset prediction, this work contributes novel insights into treatment monitoring and personalized migraine management. Crucially, the PFI analysis provided vital clinical insights by identifying medication status, patient age, and duration of study (dos) as the most influential predictors of posttreatment migraine recurrence. By elucidating the relative contribution of these clinically meaningful variables, this XAI component enhances model transparency and supports its clinical adoption. The optimized hybrid ensemble model demonstrated superior performance across all metrics, achieving an F1‐score and accuracy of 81%, with a competitive AUROC of 87%. These results validate the utility of ensemble learning for generating reliable and robust predictions in this difficult clinical domain.

Although the findings of this study are promising, the dataset used includes only 133 patients from a single biofeedback treatment program, which limits the generalizability of the models to broader populations or different treatment contexts. Future research should therefore focus on validating the proposed framework using larger, multicenter datasets encompassing diverse demographic and clinical cohorts with other validation strategies (e.g., GroupKFold). In addition, we aim to explore deep learning and transfer learning techniques to further enhance predictive accuracy. Furthermore, we intend to improve the accessibility of our study by developing a user‐friendly web‐based application to support efficient posttreatment migraine prediction.

## Author Contributions

Shibbir Ahmed Arif and Ferdib‐Al‐Islam contributed equally to this research and share first authorship.

## Funding

No funding was received for this manuscript.

## Conflicts of Interest

The authors declare no conflicts of interest.

## Data Availability

The dataset used in this study is publicly available online [19, 20].
